# Is it important to restrict head movement after Epley maneuver?

**DOI:** 10.1016/S1808-8694(15)31246-5

**Published:** 2015-10-20

**Authors:** Fernando Freitas Ganança, Ricardo Simas, Maurício M. Ganança, Gustavo P. Korn, Ricardo S. Dorigueto

**Affiliations:** Otorhinolaryngologist, Ph.D. in Medicine, UNIFESP - EPM, Affiliate Professor, Discipline of Otoneurology, UNIFESP - EPM; Resident of Otorhinolaryngology, UNIFESP-EPM; Post-graduate, Discipline of Otoneurology, UNIFESP-EPM, Full Professor in Otorhinolaryngology, UNIFESP-EPM; Post-graduation, Discipline of Otoneurology, UNIFESP-EPM; Master in Health Sciences, Post-graduation in Otorhinolaryngology and Head and Neck Surgery, UNIFESP-EPM, Post-graduate studies, Discipline of Otoneurology, UNIFESP-EPM. Study performed in the Discipline of Otoneurology, Department of Otorhinolaryngology and Head and Neck Surgery, UNIFESP

**Keywords:** vertigo, vestibular diseases - rehabilitation, nystagmus

## Abstract

The effectiveness of postmaneuver postural restrictions is controversial in patients with benign paroxysmal positional vertigo.

**Aim:**

To verify the role of postural restrictions in patients with benign paroxysmal positional vertigo of posterior canal, submitted to a single Epley maneuver.

**Study design:**

clinical prospective.

**Material and Method:**

Fifty eight patients with benign paroxysmal positional vertigo of posterior canal were randomly divided in two groups following the application of a unique Epley maneuver. The patients from group 1 were informed to restrict their head movements and to use a cervical collar and group 2 patients were not informed about these postmaneuver restrictions. The patients from both groups were reevaluated one week after Epley maneuver, regarding the presence of symptoms and positional nystagmus.

**Results:**

One week after Epley maneuver 82.1% of the patients from group 1 and 73.3% from group 2 didn't present positional nystagmus (p = 0.421). There was a clinical improvement in 96.0% of the patients from group 1 and in 94.0% from group 2 (p = 0.781).

**Conclusion:**

The use of postural restrictions in patients with benign paroxysmal positional vertigo of posterior canal didn't interfere in their clinical evaluation, one week after a unique Epley maneuver.

## INTRODUCTION

Benign paroxysmal positional vertigo (BPPV) is considered the most common peripheral vestibulopathy, present in approximately 25% of the patients that have dizziness. It is prevalent in the elderly and female patients, probably owing to senile degenerative affections and hormonal dysfunction, respectively^1^.

BPPV is caused by statoconia debris from the utriculus macula that deviate to one or more semicircular ducts, mistakenly stimulating the ampullary crest2., 3., 4..

Vertigo is usually brief - normally it lasts less than one minute, it is episodic and characteristically it is a result of the change in position of head segment. Some of the movements that caused BPPV clinical manifestations are lying down or standing up from lying down position, adopting lateral position from dorsal decubitus and head hyperextension^5^^,^^6^.

One of the main and most used therapeutic options for BPPV consists of mechanical maneuver for vestibular rehabilitation, which through a sequence of head movements, aim at repositioning the statoconia back to the utriculus7., 8., 9., 10., 11., 12., 13., 14., 15., 16., 17.. Among them, Epley maneuver, described in 1992, presents excellent therapeutic indexes of clinical improvement9., 10., 11., 12.^,^15., 16., 17..

Some authors advocate posture restriction after Epley maneuver to prevent some displacement of statoconia particles towards the semicircular duct. The patient is instructed to avoid head and trunk movement, using a neck collar and sleeping in semi-seated position, with the head inclined at 45o from the horizontal plan for two days. In the 5 subsequent days, the patient is instructed to avoid sleeping over the affected ear^9^^,^^10^^,^^12^^,^^15^^,^^17^.

The controversy in the literature is about the efficacy of posture restrictions in influencing therapeutic success in patients with BPPV submitted to statoconia repositioning maneuver^18^^,^^19^.

## OBJECTIVE

The objective of the present study was to check the importance of head movement restriction in clinical progression of patients with BPPV by duct lithiasis of the posterior semicircular canal when submitted to one single Epley maneuver.

## METHOD

The patients in this study were recruited in the Ambulatory of the Discipline of Otoneurology, Federal University of Sao Paulo - Escola Paulista de Medicina (UNIFESP - EPM) and signed the Free Informed Consent Term.

All patients presented diagnostic hypothesis of BPPV, specifically owed to duct lithiasis of posterior semicircular canal. The subjects presented typical clinical history of this vestibulopathy, comprising severe positional vertigo, with duration below one minute, which could be followed by neurovegetative symptoms, but no auditory symptoms. Physical examination of these patients revealed the presence of positioning nystagmus with rotation and upper vestibular component towards the tested ear, at Dix-Hallpike 20 diagnostic maneuver with the use of Frenzel lenses^18^.

Exclusion criteria were presence of other concomitant vestibulopathies, cervical spine affections or other reasons that prevented the performance of Dix-Hallpike and/or Epley maneuver, patients that took drugs that could influence the vestibular system.

All enrolled patients were submitted to otoneurological assessment that included clinical history, ENT physical examination, pure tone and vocal audiometry, immittanciometry and vestibular exam.

Modified Epley maneuver was performed at the diagnosis of BPPV, immediately after diagnostic confirmation using the Dix-Hallpike maneuver. Figures 1 to 3 evidence Epley maneuver, performed after Dix-Hallpike maneuver^9^.

The patients were randomly divided into two groups, according to the treatment used: Group 1 (submitted to Epley maneuver followed by head movement restrictions), and Group 2 (submitted to Epley maneuver without restrictions after the maneuvers).

Patients in Group 1 were instructed to prevent head and trunk movement, use cervical collar and sleep in a semi-seated position, with the head inclined 45o from the horizontal plan for two days. In the five subsequent days, the patient was instructed to avoid sleeping over the affected ear.

One week after the Epley maneuver, the patients in Groups 1 and 2 came back to the clinical reassessment and underwent clinical history again and Dix-Hallpike diagnostic maneuver. The assessments were performed by the examiners that did not know about group assignment.

Patients were subjectively classified as to clinical progression as cure (asymptomatic), partial improvement and no improvement (unaltered or worsened clinical presentation).

The objective assessment was performed by repeating Dix-Hallpike maneuver, one week after Epley maneuver and checking the presence of absence of vertigo and/or positioning nystagmus.

The statistical analysis were performed using chi-square test to check whether there was statistically significant difference in clinical progression (presence of positioning nystagmus and subjective assessment) of the patients with BPPV by posterior semicircular canal duct lithiasis, submitted or not to head movement restrictions after Epley maneuver. The significance level was 0.05.

## RESULTS

Fifty-eight patients with nystagmus and positional vertigo at Dix-Hallpike maneuver were submitted to Epley maneuver. The patients' age ranged from 36 to 90 years. There was predominance of female gender and 38 women and 20 men, all Caucasian.

Group 1 comprised 28 patients and Group 2 comprised 30 patients.

Duct lithiasis was present on the right ear in 32 cases (55%) and on the left ear in 26 cases (45%), as described in Table 1.

**Table 1:** Distribution of patients with BPPV, according to positioning nystagmus, pathophysiology and affected side.

Positioning Nystagmus	Pathophysiological Substrate
Upward vertical and anti-clockwise rotation (< 1 minute) with the head tilted to the right	Duct lithiasis of CPD (N = 32)
Upward vertical and clockwise rotation (< 1 minute) with the head tilted to the left	(Duct lithiasis CPE (N = 26)

Key: CPD: right posterior canal

CPE: left posterior canal.

As to detection of nystagmus at Dix-Hallpike maneuver, performed one week after treatment, we observed 82.1% improvement in patients in group 1 and in 73.3% of Group 2. There was no statistically significant difference between the 2 groups (p = 0.421).

Concerning subjective clinical progression, 95% of the total of patients presented improvement, and 60% became asymptomatic. Clinical partial and total improvement was obtained by 96.0% of the patients in Group 1 and 94% of patients in Group 2, without statistically significant different, as demonstrated in Table 2.

**Table 2:** Subjective clinical assessment of patients after one week of Epley maneuver with and without head movement restrictions.

Clinical Progression	With restrictions	Without restrictions	Total
**Asymptomatic**	18 (64,0%)	17 (56,7%)	35 (60,3%)
**Improved**	9 (32,0%)	11 (36,7%)	20 (34,5%)
**Unaltered**	1 (4,0%)	2 (6,6%)	3 (5,2%)
**TOTAL**	**28**	**30**	**58 (100,0%)**

p = 0,781

## DISCUSSION

BPPV is a high prevalence disease, usually under diagnosed. The application of an efficient treatment is important to control symptoms. One of the main and most used therapeutic options for BPPV consists of mechanical maneuvers of vestibular rehabilitation. Among them, Epley maneuver, described in 1992, is considered popular and presents clinical improvement therapeutic index9., 10., 11., 12.^,^15., 16., 17..

In this group, 58 patients with BPPV were treated by Epley maneuver. Randomly, two groups were formed and they were differentiated by the application or not of head movement restrictions and use of cervical collar after therapeutic maneuver application.

The objective of study was to assess the efficacy of use of these clinical improvement restrictions.

The age of patients presented similar variation similar to the studies by Fife et al.^21^ and Weider et al.^22^ in which ages ranged between 25 to 84 years.

Similarly to Weider et al.^22^ and Wolf et al.^23^, we also found predominance of female gender in relation to male gender. Hormonal affections could have favored higher occurrence of BPPV in women^24^.

In the present study, affection of the right labyrinth was more frequent than in the left, similarly to the results pointed out by studies by Ganança et al.^5^, Frazza et al.^25^ and Gans et al.^18^. The authors argued that the higher prevalence in the right labyrinth occurred because the diagnostic maneuver is normally started on this side and did not suffer any influence of fatigue to the repetition of the diagnostic test.

The objective of the head restrictions is to prevent incorrect displacement of statoconia or their debris after therapeutic maneuver. The period without head movement would facilitate the absorption or adhesion of statoconia to utriculus otolithic membrane.

Head movement restrictions may cause discomfort to the patient with restrictions to daily life activities and it is wondered whether their use would imply therapeutic improvement. Thus, it is important to detect the efficacy of head movement limitations after Epley maneuver, so that we can justify the clinical applications.

Gans et al.^18^ noticed that avoiding moving the head or lying down in supine position for 24 hours after Epley maneuver proved to be enough to prevent recurrences in patients with VPBB. According to Zucca et al.^26^, restrictions of head movement would not be that important after the first 24 hours after Epley maneuver, because under normal volume conditions and normal endolymph calcium content, statoconia would be dissolved within 5 to 20 hours. Experimental studies proved that in endolymphatic hydropsy, concentration of calcium in the endolymph is normally increased and that absorption of statoconia in the endolymph is inversely proportional to calcium concentration. Thus, patients with BPPV resulting from or simultaneous to endolymphatic hydropsy could be benefited by the restrictions to head movement. We emphasize the importance of new studies with the use of head restrictions in patients with associated BPPV and endolymphatic hydropsy.

In the present study, the use of head movement restrictions did not change the clinical progression of patients submitted to Epley maneuver, neither objectively, by observing positioning nystagmus. These results are in accordance with the studies by Gordon and Gadoth^27^ who detected that restrictions of head movement were not necessary for good clinical progression of patients with BPPV, submitted to Epley maneuver modified by Marciano and Marcelli^28^, who treated their patients with BPPV using Epley and Semont therapeutic maneuvers and also Nuti et al.^29^, who treated their patients with VPPV Semont therapeutic maneuvers.

The assessment of patients was made one week after Epley maneuver and did not provide information about long-term recurrence of BPPV. Therefore, we suggest the conduction of a new study whose objective is long-term clinical follow-up of patients with BPPV submitted to Epley maneuver so that we may answer this question.

## CONCLUSION

The use of head movement restrictions did not interfere in the clinical progression of patients with BPPV by duct lithiasis of the posterior semicircular canal submitted to a single Epley maneuver.

## References

[1] Ganança MM, Caovilla HH, Munhoz MSL, Silva MLG, Frazza MM, Ganança FF (1999). As muitas faces da vertigem posicional. Atual Geriatr.

[2] Hamid MA. Cupulolithiasis versus canalolithiasis: a new hypothesis. Abstract. presented at the ANS Society Meeting, Scottsdale, AZ, May, 1997.

[3] Herdman SJ, Tusa RJ., Herdman SJ (2002).

[4] Schuknecht HF. (1969). Cupulolithiasis. Arch Otolaryngol..

[5] Ganança FF. Da rotação cefálica ativa na vertigem postural paroxística benigna. São Paulo, 1999. 83p. [Tese de doutorado em medicina. Universidade Federal de São Paulo - Escola Paulista de Medicina].

[6] Herdman SJ, Tusa RJ. (1999).

[7] Brandt T, Daroff RB. (1980). Physical therapy for benign paroxysmal positioning vertigo. Arch Otolaryngol.

[8] Cawthorne T. (1944). The physiological basis of head exercises. J Chart Soc Physiother.

[9] Epley JM. (1992). The canalith repositioning procedure: for treatment of benign paroxysmal positional vertigo. Otolaryngol Head Neck Surg.

[10] Herdman SJ., Baloh RW, Halmagyi GM (1996).

[11] Herdman SJ. (1997 Jun). Advances in the treatment of vestibular disorders. Phys Ther.

[12] Herdman SJ, Tusa RJ, Zee DS, Proctor LR, Mattox DE. (1993). Single treatment approaches to benign paroxysmal positional vertigo. Arch Otolaryngol Head Neck Surg.

[13] Semont A, Freyss G, Vitte E. (1998). Curing the benign paroxysmal positional vertigo with a liberatory maneuver. Adv Otorhinolaryngol.

[14] Semont A, Sterkers JM. (1988). Reeducation vestibulaire. Cahiers Oto-rhinolaryngologie.

[15] Telian SA, Shepard NT. (1996). Update on vestibular rehabilitation therapy. Otolaryngol Clin N Am.

[16] Ganança MM, Caovilla HH., Ganança MM (1998).

[17] Ganança MM, Caovilla HH, Ganança FF. (1996). O tratamento da vertigem no idoso, por meio de exercícios vestibulares. Atual Geriatr.

[18] Gans RE, Harrington-Gans PA. (2002). Treatment efficacy of benign paroxysmal positional vertigo (bppv) with canalith repositioning maneuver and Semont liberatory maneuver in 376 patients. Semin Hear.

[19] Parnes LS, Agrawal SK, Atlas J. (2003). Diagnosis and management of benign paroxysmal positional vertigo (bppv). CMAJ.

[21] Fife TD. (1998). Recognition and management of horizontal canal benign positional vertigo. Am J Otol.

[22] Weider DJ, Ryder CJ, Stram JR. (1994). Benign paroxysmal positional vertigo: analysis of 44 cases treated by the canalith repositioning procedure of Epley. Am J Otol.

[23] Wolf JS, Boyev KP, Manokey BJ, Mattox DE. (1999). Success of the modified Epley maneuver in treating benign paroxysmal positional vertigo. Laryngoscope.

[24] Guzman PV, Zeigelboin BS, Hassan SE, Frazza MM, Diniz J, Caovilla HH. (2000). A manobra de Brandt-Daroff modificada na vertigem postural. Acta Awho.

[25] Frazza MM, Caovilla HH, Ganança MM, Cabete CF, Munhoz MSL, Silva MLG. (2001). Da direção do nistagmo de posicionamento na vertigem paroxística postural benigna. Acta Awho.

[26] Zucca G, Valli S, Valli P, Perin P, Mira E. (1998). Why do benign paroxysmal positional vertigo episodes recover spontaneously?. J Vest Res.

[27] Gordon CR, Gadoth N. (2004). Repeated vs. single physical maneuver in benign paroxysmal positional vertigo. Acta Neurol Scand.

[28] Marciano E, Marcelli V. (2002). Postural restrictions in labyrintholithiasis. Eur Arch Otorhinolaryngol.

[29] Nuti D, Nati C, Passali D. (2000). Treatment of benign paroxysmal positional vertigo: no need for post-maneuver restrictions. J Otolaryngol.

